# Repurposing old carbon monoxide-releasing molecules towards the anti-angiogenic therapy of triple-negative breast cancer

**DOI:** 10.18632/oncotarget.26638

**Published:** 2019-02-01

**Authors:** Malamati Kourti, Andrew Westwell, Wen Jiang, Jun Cai

**Affiliations:** ^1^ Cardiff China Medical Research Collaborative, School of Medicine, Cardiff University, Heath Park, Cardiff, CF14 4XN, UK; ^2^ School of Pharmacy and Pharmaceutical Sciences, Cardiff University, Cardiff, CF10 3NB, UK

**Keywords:** triple-negative breast cancer (TNBC), angiogenesis, breast cancer, carbon-monoxide releasing molecules (CORMs), anti-angiogenic therapy

## Abstract

Triple-negative breast cancer (TNBC) is defined by the lack of expression of the oestrogen and progesterone receptors and HER-2. Recently, carbon monoxide (CO) was found to behave as an important endogenous signalling molecule and to suppress VEGF receptor-2 (VEGFR-2) and protein kinase B phosphorylation. Given that anti-angiogenic drugs exist as one of the few available targeted therapies against TNBC, the aim of this project was to study the effects of CO-releasing molecules (CORMs) on TNBC cell lines and the associated endothelial cells and characterise their anti-angiogenic properties that can be used for the reduction of cancer-driven angiogenesis. Four commercially available CORMs were screened for their cytotoxicity, their effects on cell metabolism, migration, VEGF expression, tube formation and VEGFR-2 activation. The most important result was the reduction in VEGF levels expressed by CORM-treated TNBC cells, along with the inhibition of phosphorylation of VEGFR2 and downstream proteins. The migration and tube formation ability of endothelial cells was also decreased by CORMs, justifying a potential re-purposing of old CORMs towards the anti-angiogenic therapy of TNBC. The additional favourable low cytotoxicity, reduction in the glycolysis levels and downregulation of haem oxygenase-1 in TNBC cells enhance the potential of CORMs against TNBC. In this study, CORM-2 remained the most effective CORM and we propose that CORM-2 may be pursued further as an additional agent in combination with existing anti-angiogenic therapies for a more successful targeting of malignant angiogenesis in TNBC.

## INTRODUCTION

Cancer deriving from the mammary gland (breast cancer) affects more than 1.3 million women worldwide each year, with more than 55,000 reported cases in the U.K. in 2015 (Cancer Research UK). The increasing occurrence, complexity and heavy economic burden of the required treatment to the overall health system expenditure make breast cancer one of the most urgent health issues in our society [1, 2]. Although most types of breast cancer express hormone receptors (HR), there is a distinct subtype called triple-negative breast cancer (TNBC) that lacks the immunohistochemical expression of oestrogen receptor (ER), progesterone receptor (PR) and the HER2/neu protein or amplification of the HER2/neu gene. Clinical data reveal that approximately 15% of all breast cancers are diagnosed as TNBC, which occurs more frequently among minority (black race, Hispanic ethnicity) and younger women (<40 years old) and is characterized by high histological grade, higher risk of distant recurrence and shorter disease-free survival than ER-positive subtypes, alongside a more frequent metastasis to bone, lung and brain [3–5].

This unique subtype of breast cancer has yet no established effective therapeutic regimens, and the most frequently prescribed drugs are a combination of chemotherapeutic agents and receptor tyrosine kinase (RTK) inhibitors. However, the results remain disappointing and lack long-term effectiveness [6]. Angiogenesis is the multi-step process of new capillary formation from pre-existing blood vessels and plays a pivotal role in physiological processes, but it has also been recognized as a prerequisite for tumour growth, progression and metastasis [7, 8]. The angiogenic process has recently emerged as a promising target for TNBC, where it seems to be significantly over-activated and supports the growth and metastasis of the primary tumour [9, 10]. It is well established that one of the main growth factors regulating angiogenesis is the vascular endothelial growth factor (VEGF), upon interaction with its principal receptor VEGF-receptor-2 (VEGFR2) [11, 12]. VEGF itself is abundantly secreted by breast cancer cells in order to promote differentiation and aggressive phenotypes. By overexpressing VEGF, TNBC cells succeed in stimulating angiogenesis conducted by the adjacent vascular endothelial cells (ECs) and providing further nourishment for the growing tumour, the remove of the waste as well as the entering point of the distinct metastasis. Therefore, targeting the interaction between VEGF and VEGFR2, as well as other downstream kinases might not only help in developing better therapies for TNBC, but also overcome the chemo- and radiation resistance correlated with TNBC [13]. Since recent investigations into the use of anti-VEGF therapies such as bevacizumab alongside chemotherapy have ultimately proved ineffective in the long-term for patients with invasive breast cancer, and given the status of anti-VEGF drugs as one of the few available targeted therapies for TNBC, there remains an urgent and unmet medical need for improving the existing anti-angiogenic therapies [14].

For a long time, carbon monoxide (CO) has been best known for its toxic effect as an air pollutant because of its strong affinity (>220-fold greater than that of oxygen) for the iron of haemoglobin. However, endogenously produced CO by haem oxygenases (HO) was found to regulate various molecular signalling pathways related, among others, to cellular metabolism, and was therefore characterized a gasotransmitter [15, 16]. However, since the administration of CO gas can be dangerous and poorly regulated, the discovery of transition metal carbonyls that can act as CO-releasing compounds (CORMs) has provided a safer way to control CO release *in vivo* in a spatial and temporal manner [17]. Previous studies suggested that CO can have various and even opposite effects on different subtypes of cancer and CORMs might act as a new therapeutic entity [18–20]. Additional to the observed heterogeneous effects on different systems and cell types, recent research has also demonstrated that the ruthenium-based first generation CORM-2, can exhibit anti-angiogenic activity by inhibiting VEGF-induced EC functions and the activation of VEGFR2 [21].

Based on the above observations, we designed this study to shed more light on potential anti-angiogenic properties of CORMs in a TNBC subset. Since CORM-2 has been thoroughly tested in various cell models with contradicting results, three other first and second generation CORMs were also tested in our study and compared with CORM-2. Here we reported the novel observations of the anti-angiogenic activity of CORMs that will eventually help in elucidating the mechanism of action of these unique organometallic complexes as anti-angiogenic agents. In the term of anti-angiogenic activity, CORM-2 remained the most effective CORM and we propose CORM-2 as a potential agent to be pursued further in combination with existing anti-angiogenic therapies for a more successful targeting of malignant angiogenesis in TNBC.

## RESULTS

### CORMs reduce VEGF expression in TNBC cells

In order for tumour angiogenesis to take place, two important steps must be accomplished. First, the tumour cells should be able to express and secret high concentrations of VEGF and other growth factors, in order to induce pro-angiogenic signals. Second, vascular ECs around the tumour area should express effective VEGFRs (RTKs) that upon binding with VEGF will initiate the pro-angiogenic signal through activation of their downstream signalling pathways. This activation cascade involves the phosphorylation of several kinases that manipulate EC survival, proliferation, migration and vessel formation. Therefore, an anti-angiogenic agent could potentially inhibit several different steps of this process, either the ability of cancer cells to express the elevated levels of the pro-angiogenic molecules or the ability of ECs to execute an efficient signal transduction that leads to new vessel formation. In that scope, CORMs were subjected to relevant experiments in order to assess their ability to affect both required steps for successful angiogenesis.

**Figure 1:**
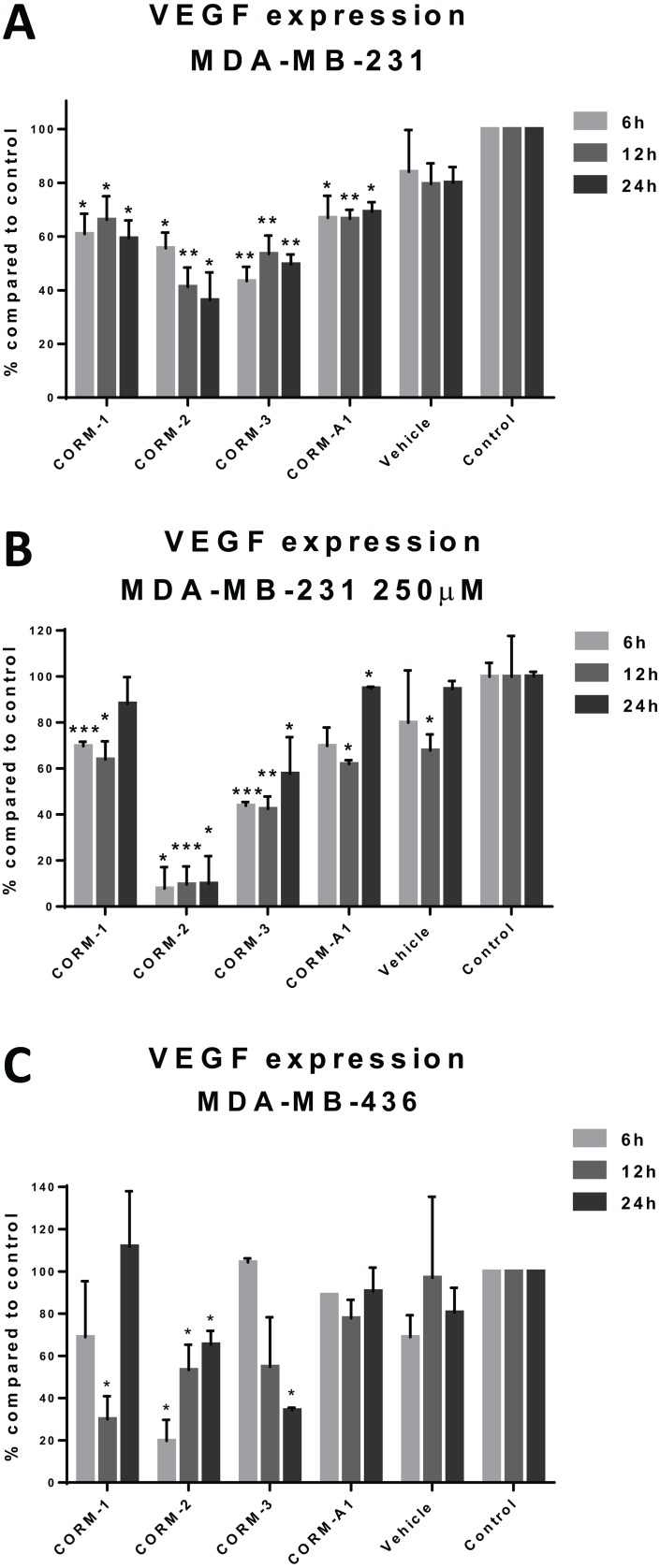
VEGF expression after CORM treatments (**A**) Quantification of VEGF levels in the supernatants of treated MDA-MB-231 cells with 100 μM CORMs or vehicle or normal media for 6 h, 12 h or 24 h. (**B**) Quantification of VEGF levels in the supernatants of treated MDA-MB-231 cells with 250 μM CORMs or vehicle or normal media for 6 h, 12 h or 24 h. (**C**) Quantification of VEGF levels in the supernatants of treated MDA-MB-436 cells with 100 μM CORMs or vehicle or normal media for 6 h, 12 h or 24 h. (% percentage compared to media treated cells (control) ±SEM; *n* = 3, *N* = 3) (All data was statistically analysed against media treated cells using unpaired *t-test* with Welch's correction: ^*^p < 0.05, ^**^p < 0.01, ^***^p < 0.001).

MDA-MB-231 cells were treated with CORMs and the concentration of VEGF excreted in the cell culture supernatant was quantified with a human VEGF ELISA kit. As observed in Figure 1A, all four CORMs reduced the concentration of excreted VEGF in MDA-MB-231 cells. More specifically, in the 6 h treatment CORM-3 reduced VEGF by more than 55% reaching high statistical significance and CORM-2 followed as the second most effective one. The reduction in the expression of VEGF in MDA-MB-231 cells remained evident even in the longer time treatments. In the 12 h treatment, CORM-2 proved the most efficient, reducing VEGF expression by 60% with the greatest statistical significance among the others, but CORM-3 remained extremely successful. Finally, in the 24 h treatment, the results were similar, with CORMs -2 and -3 reducing the concentration of the excreted VEGF by 65% and 50%, respectively, with high statistical significances. Subsequently, these CORMs were also tested at a higher concentration of 250 μM, to seek for concentration-dependent effects (Figure 1B). However, only CORM-2 appeared to reduce VEGF expression in a significantly higher level compared to the lower concentration, but this could also be a cytotoxicity-linked result, so the effect was not deemed concentration-dependent.

The other TNBC cell line involved in this study, MDA-MB-436, was also studied for similar reduction effects and the results of 100 μM treatments at three different time points are depicted in Figure 1C. CORM-2 and CORM-3 were the most successful in decreasing the excreted VEGF, although higher variations were reported for this cell line leading to lower statistical significance. CORM-2 was the only CORM that raised significantly different results compared to control at all time points. However, a similar trend was observed for both TNBC cell lines.

### CORM-2 and CORM-3 abrogate the phosphorylation of downstream proteins of the VEGFR2 signalling pathway in VEGF stimulated HUVEC cells

Within the tumour microenvironment, the VEGF excreted by cancer cells binds to its receptors on the EC surface. Upon binding, the receptor auto-dimerizes and transfers the pro-angiogenic signal within the EC, through consecutive phosphorylation of downstream kinases [22].

**Figure 2:**
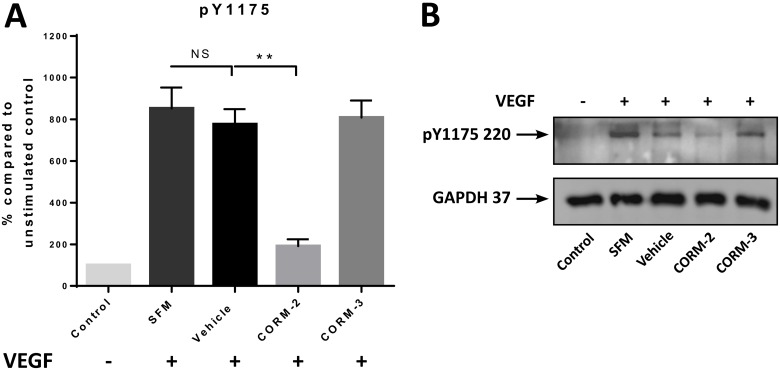
pY1175 levels after CORM treatments (**A**) Expression levels of pY1175 of VEGFR2 in HUVEC after 15 min of CORM or vehicle pre-incubation and then stimulation with VEGF (100 ng/ml) for 5 min (SFM), as measured with the human pY1175-VEGFR2 ELISA kit. The first sample is the unstimulated control (Control). (Graph shows % compared to Control ±SEM; *N* = 3) (Data statistically analysed using unpaired Student's *t-test* with Welch's correction ^*^p < 0.05, ^**^p < 0.01). (**B**) Representative blot of the levels of pY1175 of VEGFR2 following the same protocol (*N* = 3).

Since the successful downstream transfer of the angiogenic signal is vital to the vessel formation activity of ECs, we aimed to investigate the ability of the two most promising CORMs, CORM-2 and CORM-3 to inhibit the activation of some of these kinases in primary vascular ECs (HUVEC) upon VEGF stimulation. The first target, VEGFR2, has multiple phosphorylation sites, but Y1175 was chosen. Therefore, an ELISA quantification was performed for the phosphorylation levels of VEGFR2 at Y1175 upon CORM or vehicle pre-treatment and VEGF stimulation, and as shown in Figure 2A, CORM-2 managed to return the phosphorylation at the baseline levels of the unstimulated control (Control). This reduction reached high statistical significance. CORM-3 failed to decrease these levels, and the same result was also verified via a Western blot analysis, suggesting that only CORM-2 could inhibit the phosphorylation of Y1175 of VEGFR2 (Figure 2B).

**Figure 3:**
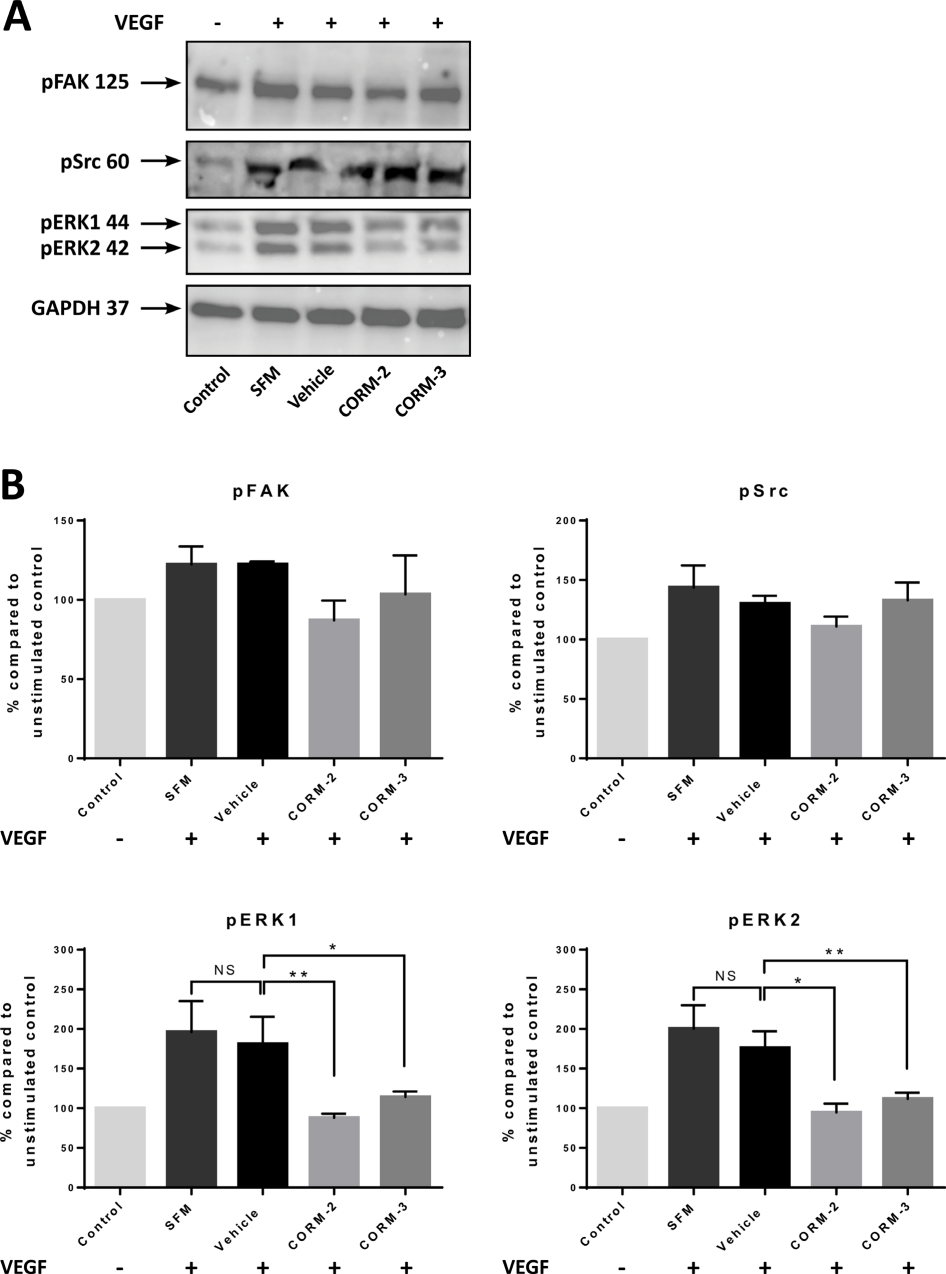
Expression of VEGFR2 pathway proteins in HUVEC after CORM pre-treatments and VEGF stimulation (**A**) Western blot of pFAK, pSrc and pERK1/2 following 15 min of CORM or vehicle pre-incubation and then stimulation with VEGF (100 ng/ml) for 5 min (SFM). The first sample is the unstimulated control (Control). (Blots show representative data; *N* = 3). (**B**) Quantitative assessment of pFAK, pSrc and pERK1/2 levels in HUVEC. (Graphs show % compared to Control ±SEM; *N* = 3) (Data statistically analysed using nonparametric (Mann-Whitney) *t-test* with ^*^p < 0.05, ^**^p < 0.01).

Some downstream proteins were also chosen to be tested via Western blot analysis. As shown in Figure 3A and 3B, both CORM-2 and CORM-3 significantly downregulated the phosphorylation of ERK1 and ERK2 at baseline levels. On the contrary, the phosphorylation of Src at Y419 was not significantly inhibited by the two CORMs, although the stimulation achieved with this protocol was very moderate for Src (measured increase of the phosphorylation by ~50% for the control and ~25% for the vehicle compared to the Control) (Figure 3B). Finally, FAK was also mildly stimulated by VEGF leading to no statistical significance for the reduction in the phosphorylation levels of Y397 produced by CORM-2, but the trend of downregulation was again observed (Figure 3B).

### Reduction in VEGF expression by TNBC cells leads to lower tube formation ability of ECs

As outlined thoughout this work, angiogenesis is central to many normal physiological processes, but has also been linked to tumour growth, progression and metastasis [7, 23]. The prevalent form of pathological angiogenesis is sprouting angiogenesis, which participates in tumour neovascularization [11].

**Figure 4:**
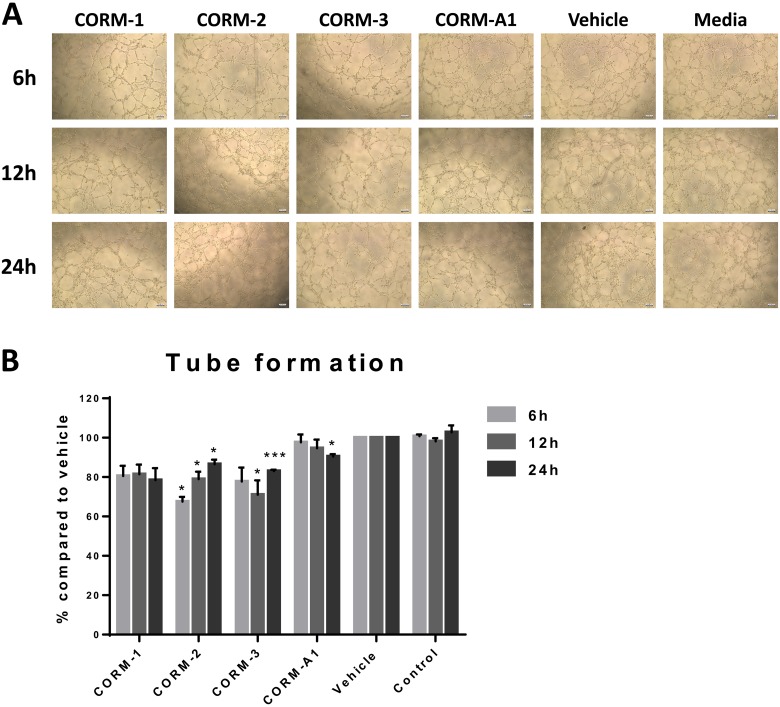
Tube formation ability of HECV after conditioned media treatments (**A**) Representative images from tube formation assay with HECV cells treated with conditioned media from variable duration incubation of MDA-MB-231 cells with 100 μM CORMs or vehicle or normal media. Objective 5x, Scale bar = 132.08 μm. (**B**) Quantification of tube formation capacity of HECV cells after treatment with conditioned media. (% Total tube perimeter compared to vehicle ±SEM; *n* = 3, *N* = 3) (All data was statistically analysed against the conditioned media from corresponding duration vehicle treated MDA-MB-231 cells using un-paired *t-test* with Welch's correction: ^*^p < 0.05, ^**^p < 0.01, ^***^p < 0.001).

Since VEGF is the major mediator of angiogenesis and the interaction between VEGF and its receptors facilitates tumour growth especially in TNBC, we, therefore, studied the CORM-mediated effect of the reduction in VEGF expression from TNBC cells on the tube formation ability of ECs. Conditioned media from CORM-treated MDA-MB-231 cells were used to stimulate tube formation in HECV cells. As shown in Figure 4A and 4B, conditioned media from CORM-1, CORM-2 and CORM-3 treatments managed to reduce the formation of tubes by ECs, with CORM-2 and CORM-3 reaching statistical significance. The tubes observed were fewer and longer compared to vehicle and control groups.

### CORM-2 directly eliminates the tube formation ability of ECs

**Figure 5:**
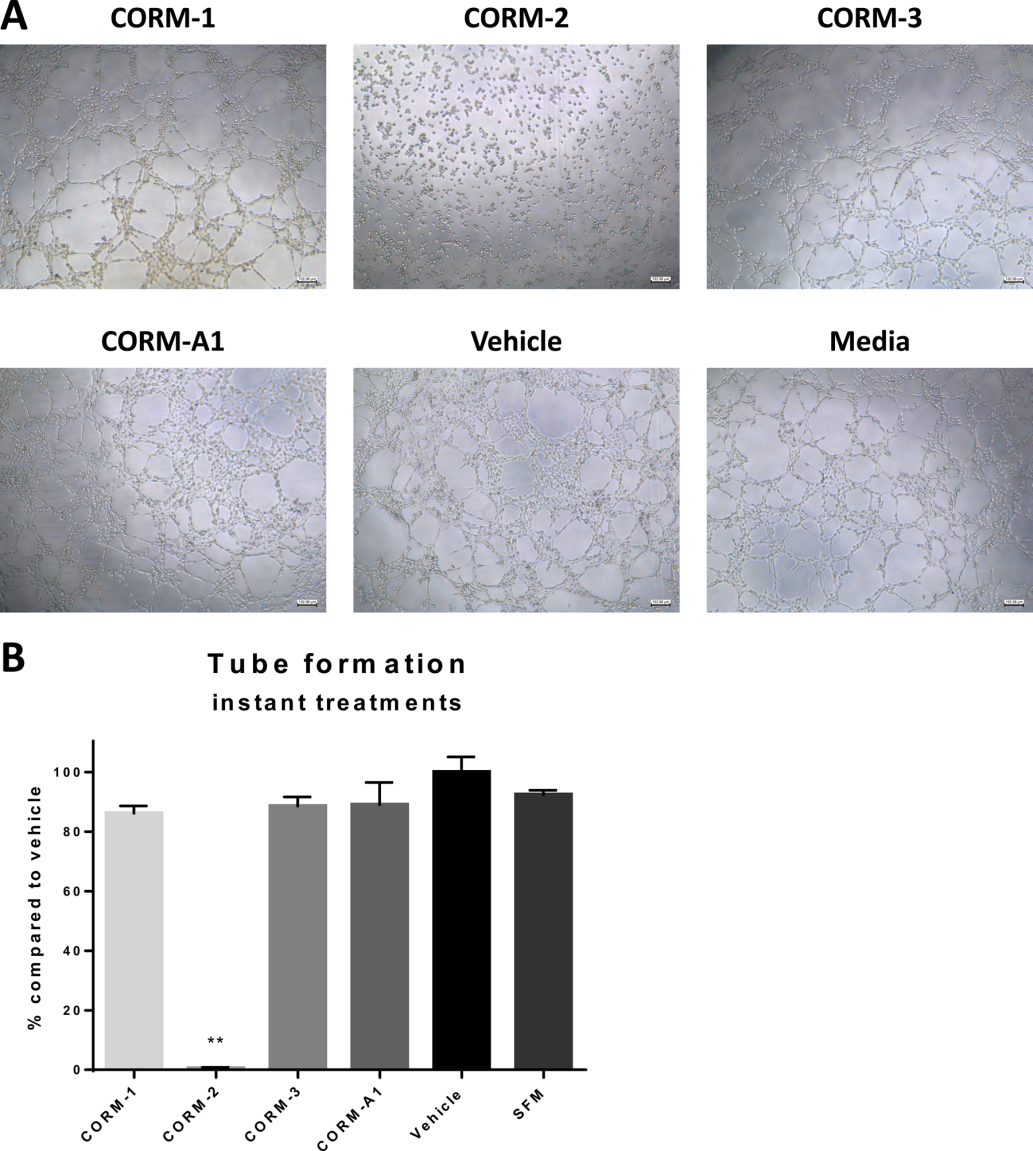
Tube formation ability of HECV after CORM treatments (**A**) Representative images from tube formation assay with HECV cells treated with 100 μM CORMs or vehicle or serum free media. Objective 5x, Scale bar = 132.08 μm. (**B**) Quantification of tube formation capacity of HECV cells after treatment with CORMs or vehicle or serum free media. (% total tube perimeter compared to vehicle ±SEM; *n* = 3, *N* = 3) (All data was statistically analysed against the vehicle group using un-paired *t-test* with Welch's correction: ^*^p < 0.05, ^**^p < 0.01).

To investigate the direct effect of CORMs on the tube formation ability of ECs, a separate experiment was conducted, where ECs were directly treated with the CORMs or vehicle or serum free media and their tube formation capability was assessed by quantifying total tube length in images taken 6 h after treatments. As shown in Figure 5A and 5B CORM-2 completely eliminated the formation of tubes with high statistical significance, whereas the other tested CORMs did not produce such a significant inhibition.

### CORMs inhibit the migration of ECs

Angiogenesis is directly linked to the capability of ECs to proliferate and migrate in response to growth factor activation. There is a fine balance between pro- and anti-angiogenic factors that determines the formation of new blood vessels, not only in a normal environment but also in a cancerous one. In this process, migration of capillary ECs plays a major role and it follows the stimulation induced by the secreted and circulating VEGF [24]. Finding agents that can disrupt the migration of ECs would be a promising alternative approach towards the inhibition of cancer-mediated angiogenesis.

**Figure 6:**
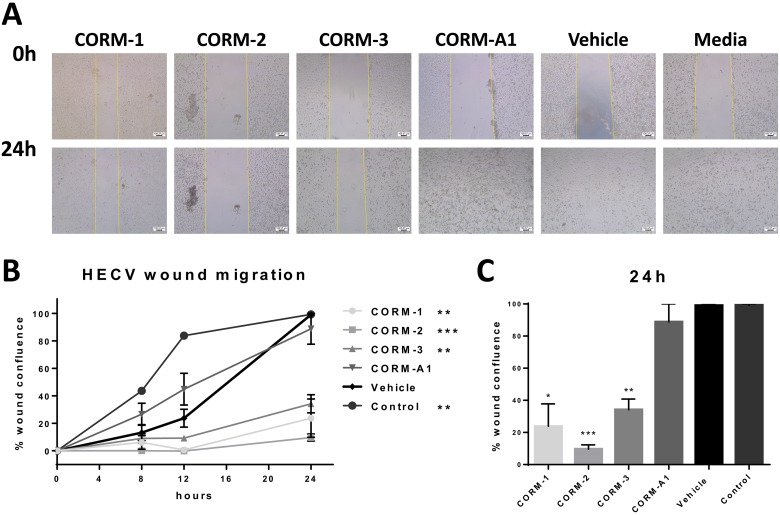
Migratory ability of HECV after CORM treatments (**A**) Representative images from a scratch wound assay in HECV cells at 0 h and 24 h after treatment with 100 μM CORMs or vehicle or normal media. Objective 5x, Scale bar = 100.37 μm. (**B**) Assessment of the healing, expressed as % wound confluence, at all time points tested after treatments. (% wound confluence ±SEM; *n* = 3, *N* = 3) (All data was statistically analysed against vehicle treated cells using two-way ANOVA: ^*^p < 0.05, ^**^p < 0.01, ^***^p < 0.001). (**C**) Average % wound confluence at the final time point (24 h) for all treatments. (All data was statistically analysed against the vehicle group using un-paired *t-test* with Welch's correction: ^*^p < 0.05, ^**^p < 0.01, ^***^p < 0.001).

For these reasons, the four available CORMs were tested for their ability to inhibit the migration of ECs following a wound formation on their monolayer. Figure 6A present the results of a traditional scratch wound assay in ECs after CORM, vehicle or media treatments. As observed, CORM-1, CORM-2 and CORM-3 blocked the migration of ECs, leaving the induced wound still open, even 24 h after the scratch (Figure 6A). This inhibition reached significance at the final time point of 24 h for all three CORMs, as calculated in an un-paired *t-test* with Welch's correction and depicted in Figure 6C. It should be noted though, that the migration of the vehicle treated cells was delayed compared to the control group, and this difference reached significance with *p* = 0.0056, as calculated in a two-way ANOVA test (Figure 6B). However, the wound managed to close anyway after 24 h.

### CORMs express moderate cytotoxicity against TNBC, epithelial and EC lines

According to general opinion, heavy metals and drugs based on them may present toxicological concerns not only to the environment in general, but also to different cells and organisms. The same applies to organometallic compounds with different metals bound, but the toxic effects derive mainly from the intrinsic characteristics of each one, such as the oxidation state, ligand sphere or the counter-ion present [25]. CORMs are a special class of organometallic complexes, as the CO presents additional toxicological hazards that should not be overlooked.

Therefore, the two TNBC cell lines, MDA-MB-231 and MDA-MB-436, along with the MCF-10A breast epithelial cells and HECV immortalized ECs were assessed for their viability upon CORM treatments for 72 h using the MTT colorimetric assay. No major cytotoxicity compared to the vehicle was observed against these cell lines and the calculated IC_50_ values exceeded 100 μM (Supplementary Figure 1A). Only CORM-2 provoked toxicity in MCF-10A cells with a calculated IC_50_ value of 31.87 μM. Following this, the 100 μM concentration was chosen for further studies and the % percentage cell viability for each CORM and cell line at this concentration can be seen in Supplementary Figure 1B.

### CORM-2 decreases the glycolytic metabolism of TNBC cells

In 1924 Otto Warburg realized that cancer cells depend on aerobic glycolysis to metabolize glucose and generate ATP. This phenomenon is referred to as the “Warburg effect” [26]. The up to 200 times higher rate of glycolysis in cancer cells is followed by lactic acid fermentation in the cytosol, unlike normal cells which oxidize pyruvate in mitochondria [27]. This observation of high glycolysis levels of cancer cells compared to normal cells, was deemed a promising target for new anti-cancer therapies. Inhibition of glycolysis would deprive cancer cells of energy, reducing their rapid growth and proliferation [28, 29].

With this in mind, investigation into whether this metabolic modulation was evident in TNBC cells upon CORM treatments was desired and a Seahorse XF Glycolysis Stress Test was conducted. MDA-MB-231 cells were chosen due to being a highly aggressive and metastatic TNBC cell line [30, 31]. In the two concentrations tested (100 μM, 50 μM), only CORM-2 raised a statistically significant reduction in the glycolysis change rate compared to the vehicle group with a very high statistical significance for the higher concentration. Other CORMs did not show any significant effect on the glycolytic metabolism of TNBC cells (Supplementary Figure 2).

### CORMs decrease HO-1 expression in MDA-MB-231 cells

Haem oxygenase-1 (HO-1) is a well-characterised cytoprotective enzyme that defends the cell in conditions of oxidative stress and excessive ROS production [32]. CORMs have been reported to increase ROS production, especially in bacterial, but also in eukaryotic cells [33].

Therefore, this study aimed to investigate whether CORMs could potentially alter the expression of this enzyme, affecting the redox status of the cancer cells and their antioxidant defence systems. MDA-MB-231 cells were treated with 100 μM CORMs for 12 h and the expression of HO-1 was quantified via Western blot analysis. As observed in Supplementary Figure 3A and 3B, CORM-1 and CORM-2 significantly reduced the expression of HO-1 after 12 h of treatment, whereas CORM-3 failed to reach this level of significance, even though the same reducing trend was observed. On the other hand, CORM-A1 increased the expression of this enzyme by 40%, indicating a different mechanism of interaction with the HO-1 system than the other CORMs.

## DISCUSSION

The aim of this study was to screen four commercially available first and second generation CORMs for effects against TNBC. With this in mind, several experiments were conducted trying to relate these complexes to anti-angiogenic activities, such as effects on the stimulant activity of TNBC cells *per se* or on the signalling and migratory activities of ECs that are responsible for the angiogenic process.

Tumour angiogenesis refers to the highly interactive process of the formation of new blood vessels from pre-existing ones. It is linked to the expression of several proteins, both by the cancer cells themselves and by the ECs that line the surrounding blood vessels and execute the process of neovascularization [34]. A logical approach would be to combine agents that can reduce the expression of angiogenic factors with ones that target these factors *per se*, or their receptors. The principle behind this combination involves the potential enhancement of the delivery of cytotoxic agents to the tumour site, as well as the possible interference with the ability of the tumour to recover from the effects of the accompanying chemotherapeutic agent [35]. With this in mind, the next step of the screening of the four commercially available CORMs was to measure the expression of proteins related to angiogenesis and responsible for its successful accomplishment.

The main pro-angiogenic factor, VEGF, participates in the angiogenic process by increasing vascular permeability and stimulating EC survival, proliferation, migration and expression of MMPs among others [34]. Intriguingly, some reports suggested an increase in VEGF expression induced by CORMs in several *in vitro* models. More specifically, ECs seemed to stimulate their VEGF expression upon treatment with CORMs, CORM-2 and CORM-3 in most studies, and this pointed towards an increased angiogenic potential for these cells [36, 37]. Other cells showed a similar behaviour. For instance, CORM-2 caused an increase in VEGF secretion in astrocytes [38] and CORM-401 and CORM-A1 led to an enhanced expression of VEGF in microglia cells after 3 h of treatment, whereas after 6 h the levels returned back to normal [39]. However, there are also studies contradicting these results, such as the study from Ahmad et al. [21] where HUVEC – primary vascular ECs – were reported to downregulate the phosphorylation and activation of both VEGFR2 and AKT upon treatment with CORM-2, suggesting a potential anti-angiogenic ability of this compound. Nevertheless, none of these studies included cancer cells, and there are extensive reports that the effects of CO and CORMs are cell and tissue-type specific. [19] It was deemed useful to study the effects of the commercially available CORMs on the expression of VEGF from TNBC cells.

The reduction in VEGF reached a level of 50–60% in this study, suggesting a very promising profile for these complexes that can potentially halt the elevated expression of VEGF by TNBC cells, possibly also decreasing the angiogenic stimulation reaching the surrounding ECs. It would be important to mention that these results were significant, and all four CORMs seemed to share the same tendency to reduce the VEGF expression of TNBC cells in a dose-dependent manner for CORM-2. However, it should be considered whether this reduction was a direct effect of potential cellular toxicity of this compound. Thus, the main observation of this experiment was the reduction in the excreted VEGF from TNBC cells at all time points tested, leading to potentially decreased angiogenic stimulation towards ECs. These complexes were shown to interfere with the expression pathway of VEGF in TNBC cells, possibly leading to lower stimulation signals to the surrounding tissues. There are differences in the VEGF expression and regulation mechanisms between TNBC and normal breast cells. Hence, this altered mechanism might well be prone to CORM interference.

According to general opinion, heavy metals and drugs based on them may present toxicological concerns not only to the environment in general, but also to different cells and organisms. Metal toxicity, however, is tightly correlated with the form in which the metal is present, as pure elements may differ greatly from their solubility, oxidation state and bioavailability variations [25]. Organometallic compounds are a special class of molecules, especially CORMs that are able to release CO, and a toxicological investigation should be conducted before conclusions about their activities can be safely reached.

In the assessment of cytotoxicity for the four commercially available CORMs the two TNBC cell lines, MDA-MB-231 and MDA-MB-436, along with the control epithelial breast cell line, MCF-10A, and the vascular EC line, HECV, were tested. These compounds exerted a moderate cytotoxicity against the cell lines, with the most potent compound in general being CORM-2. As found in previous studies, CORM-2 was toxicity against different cell subtypes at 50 μM [21, 40, 41]. The toxicity of CORM-2 against ECs along with TNBC cells might be desired, but the quite high toxicity against breast epithelial cells is definitely a drawback. However, Ru compounds are generally believed to cause fewer and less severe side effects compared to other organometallic drugs, and they have shown broad diversity in their activity, toxicity and mechanisms of action [42–44].

CORM-3 on the other hand, even though sharing many common features with CORM-2, such as the Ru core and the similar ligand sphere, exerted a considerably lower toxicity against the tested cells. It appeared to be more potent against MDA-MB-231, and almost no cytotoxicity was reported against epithelial or ECs, suggesting a kind of selectivity for this CORM.

CORM-1 was the other first generation CORM tested, which also belongs to the photo-CORM family. We observed that the two TNBC cells were more sensitive compared to the epithelial or endothelial ones. Finally, CORM-A1 did not show any cytotoxicity against the tested cells and it is also the only one without a heavy metal core. Therefore, the metal core may be partly responsible for the moderate toxicity associated with CORMs -1, -2 and -3, although no major cytotoxicity was reported for any of the cells involved. This is also an indication that the heavy metal core is not necessarily toxic in all potential forms, and that other characteristics may affect this behaviour.

Another important aspect of angiogenesis is the successful pro-angiogenic signal transduction from the surface receptor VEGFR2 to its complex network of downstream proteins [7]. VEGF has a higher affinity for VEGFR1, but the effects of activation are much more profound for VEGFR2, regulating EC functions [12]. VEGFR2 has also been found upregulated in TNBC [9, 10, 45]. As an RTK, VEGFR2 undergoes dimerization and oligomerization, which results into auto- and trans-phosphorylation on specific tyrosines in the cytoplasmic domain. Two of the most important tyrosine residues (autophosphorylation sites) are Y1175 and Y1214 [46, 47]. Therefore, CORMs were studied for their ability to reduce the phosphorylation of one of these tyrosine residues of VEGFR2, in order to investigate any inhibitory activity on the activation of this major receptor. Intriguingly, we demonstrated that only CORM-2 appeared to inhibit the activation of this receptor on Y1175, reducing the phosphorylation almost to baseline levels in a statistical manner, whereas CORM-3 failed to produce such an inhibition, pointing towards a very promising behaviour of CORM-2. This was a particularly interesting finding that correlates well with previous literature [21, 48] and suggests an impairment of the angiogenic signal transduction after treatment with CORM-2, even upon stimulation with a high dose of VEGF that is far higher than the normal VEGF concentrations in the tumour microenvironment [49].

As a proof of concept, more downstream proteins were investigated upon stimulation with VEGF, in order to shed light on any potential targets of CORM-2 and CORM-3 [34]. Different studies reported contradictory results. The work done by Otterbein et al. [50] and Brouard et al. [51] suggested an upregulation of pERK1/2 in stimulated macrophages, fibroblasts and ECs upon treatment with CO gas, whereas Song et al. [52] showed an inhibition of ERK phosphorylation in stimulated human gingival fibroblasts upon treatment with CORM-3. Taillé and colleagues [53] found decreased ERK1/2 phosphorylation in airway smooth muscle cells upon treatment with CORM-2, which implicated ROS production. Therefore, we studied HUVEC cells for the phosphorylation levels of different proteins of the VEGFR2 pathway upon VEGF stimulation and found that CORM-2 and CORM-3 exerted a differential inhibitory activity. For example, CORM-2 was effective in inhibiting the phosphorylation of all tested proteins reaching significance for ERK1/2. Even though a similar downregulating activity was observed also for Src and FAK proteins, this did not reach significance probably due to the quite mediocre activation of these proteins under the given conditions. CORM-3, on the other hand, was shown to be preferentially effective against ERK1/2. This suggested a different mechanism of action for the two CORMs of similar chemical structure, but anyway confirmed their potential as anti-angiogenic agents.

Commercially available CORMs seemed to induce a modest reduction in the levels of VEGF expressed by TNBC cells. Following this, it was speculated that these decreased levels of growth factor would have a subsequent decreasing effect on the ability of ECs to form tubes. Conditioned media from CORM treated TNBC cells stimulated angiogenesis at a lower level compared to vehicle or media treated cells, suggesting a potential inhibition of the capability of ECs to form tubes. CORM-2 had the most profound effect and the second best CORM was CORM-3.

As CORM-treated TNBC conditioned media showed less angiogenic stimulation than non-treated conditions, we asked a question whether CORMs possess a direct effect on the angiogenic functions of ECs. When a tube formation experiment was conducted with direct CORM treatments on ECs, CORMs -1, -3 and -A1 did not reduce tube formation in a significant manner, suggesting a minimal interference with the ECs themselves. However, CORM-2 treatment surprisingly reduced tube formation completely and further analysis revealed that CORM-2 treated ECs remained stable and were unable to migrate and create capillary-like formations. Our migration results confirmed the notion that CORM-2 interfered with the ECs themselves and correlated well with the inhibition on migration previously shown for this compound [21]. This result suggested a strong anti-angiogenic behaviour of CORM-2 when in direct contact with ECs and should not be linked to the moderate toxicity of this compound towards HECV cells.

All these observations lead to a preliminary conclusion that these compounds and especially CORM-2 can act anti-angiogenically. It is documented that other VEGFR inhibitors and monoclonal antibodies that target angiogenesis have generally failed to show significant overall efficacy in the clinic [54], therefore new drugs that can combine different mechanisms of action are currently pursued. CORMs could be such a drug, benefiting the anticancer therapy from many points, and as a part of a combination treatment enhance the activity of existing drugs such as other VEGFR or VEGF-A inhibitors. By combining the VEGF-decreasing activity with the VEGFR activation inhibition, CORMs can also help in the reduction of the dosage for existing anti-angiogenic drugs or lead to the reuse of revoked drugs for breast cancer, such as Avastin due to the non-significant improvement of patient survival and several side effects [55–57]. Limiting these side effects could lead to more effective and less harmful combination therapies that address malignant angiogenesis in many ways, also reducing the risk of resistance.

To expand the investigation of CORMs against TNBC, some other effects were also studied. These tests aimed to indicate other anti-TNBC properties of CORMs related to important cell functions such as viability, metabolism and HO-1 expression, which is a crucial cytoprotective enzyme.

Cancer has long been linked to mitochondrial dysfunctions. Aerobic glycolysis (Warburg effect) can be more efficient in supporting the rapid growth of cancer cells, compared to normal glycolysis linked to the TCA cycle and oxidative phosphorylation. In addition, diminishing mitochondrial activity offers cancer cells a way to reduce apoptosis, therefore provides an evolutionary advantage. The reprogramming of cellular metabolism and the shift of the environmental pH towards the acidic region during malignant transformation suppresses the growth of normal cells and potentially supports cancer cell growth and migration [29, 58].

CO has been shown to exert a variety of effects on mitochondrial function and cell metabolism, depending on its concentration, the duration of exposure and the specific cell subtype studied [59]. It is generally accepted that CO can inhibit cellular mitochondrial respiration via inhibition of complex IV (cytochrome c oxidase). Even though low concentrations can activate survival pathways, high concentrations of CO can increase mitochondrial oxidative stress, inhibit mitochondrial electron transport chain and protein synthesis and maybe activate apoptotic pathways in predisposed cells [60–65]. It has been shown that exogenous CO reduces glucose consumption as well as increases oxygen consumption, indicating a CO-induced improvement in oxidative phosphorylation levels [59]. CO can also limit prostate cancer progression by manipulating cell metabolism, thus sensitizing cancer cells to chemotherapy via promoting an anti-Warburg effect. Indeed, CO can target the mitochondrial activity, inducing higher oxygen consumption levels and free radical generation, thus leading to mitochondrial collapse, cancer cell growth inhibition and maybe apoptosis, induced potentially by chemotherapy [66]. Finally it has been demonstrated that CO released from a novel CORM (CORM-401) induced uncoupling of mitochondrial respiration and repression of glycolysis, which could lead to the inhibition of pathological angiogenesis [67].

CORMs were tested for their effect on the glycolysis levels of MDA-MB-231 cells. It has been previously shown that CO release from low concentrations of CORM-3 interact with the mitochondrial respiratory chain by uncoupling proteins and also with adenine dinucleotide transporters leading to disruption of the membrane potential. Ruthenium compounds have demonstrated significant mitochondrial toxicity suggesting that the Ru core might also be responsible for the impairment of complex I and IV activity [68, 69]. Therefore, our results could be explained by supporting the hypothesis that the Ru core of CORM-2 along with the CO reduce the glycolytic metabolism of TNBC cells by interfering with the mitochondrial activity, inhibiting proteins of the respiratory chain and leading to electron leakage and ROS production.

It has been previously shown that suppression of HO-1 expression can impede the proliferation and viability of pancreatic cancer cells [70], as well as the survival and growth of hepatocellular carcinoma [71] and prostate cancer cells [72]. However, in other studies HO-1 silencing was oppositely reported to increase tumour growth [73], pointing towards a rather complex and tumour type-specific role. Most of the increasing evidence suggest a link between lower expression of HO-1 and higher sensitivity of cancer cells [65]. A study done by Taillé et al. [53] suggested no change in HO-1 expression after 24 h treatment with up to 10 μM CORM-2 in airway smooth muscle cells but shorter treatments against TNBC cells have not been explored according to our knowledge. Our results showed a downregulating activity of CORMs towards HO-1 that was significant for CORMs -1 and -2 but failed to reach significance for CORM-3. However, the pattern observed for these complexes was not shared by CORM-A1, which markedly increased HO-1 expression in TNBC cells. These observations suggest two different concepts: 1) that transition-metal based CORMs can decrease the expression of HO-1 in TNBC, a mechanism not followed by the non-transition metal-based CORM-A1, and 2) that this downregulation might lead to a higher sensitivity of these cells towards other chemotherapeutic or anti-angiogenic agents, based on previous observations for other tumour types [70].

Nevertheless, it seems that CORM-2 is the best analogue of the four CORMs we tested, pointing towards a mechanism of action targeting the VEGF expression in TNBC cells and the activation of ERK1/2 and other proteins in ECs. CORM-2 was also shown to reduce the glycolytic metabolism of TNBC cells, downregulate HO-1 and inhibit EC migration. Tube formation was significantly reduced after treatment with conditioned media from CORM-2-treated cancer cells and eliminated completely after direct treatment with this CORM. The observed abilities probably cannot be solely attributed to the released CO, otherwise a very similar effect would have been reported for all tested CORMs since they are reported to release comparable amounts [74–78]. Our hypothesis is that the Ru core might also play an important role in the observed results, as well as the dimeric form of CORM-2 that can potentially enhance interactions with cellular targets. Most studies use CO-depleted molecules as negative controls for their experiments and indeed find a significantly decreased efficacy upon CO elimination [21, 41]. On the other hand, in our study very similar molecules such as CORM-2 and CORM-3 showed various differences in their actions, determining CORM-2 as a more effective compound. Therefore, we firmly believe that the results reported here depend both on the metal centre and the released CO, but a future more in-depth study remains to determine the true contribution of each factor by comparing Ru-based molecules without CO ligands and Ru-based CORMs with different ligands than in CORM-2 and CORM-3.

In summary, ongoing experiments remain to shed more light on the specific cellular targets that relate CORM-2 to the VEGF expression mechanism in TNBC cells and to specific proteins in the VEGFR2 pathway of ECs. Based on these observations though, CORM-2 can be suggested for a holistic approach against malignant angiogenesis in TNBC, due to its interference with several steps of the angiogenic process and the metabolism of TNBC cells. When a more detailed mechanism of action is discovered for CORM-2, several combination therapies can be explored that will be able to target vital components of malignant angiogenesis succeeding in eliminating it.

## MATERIALS AND METHODS

### Reagents

Recombinant human VEGF-A was purchased from R&D Systems (Minneapolis, USA). We obtained the mouse anti-phospho-ERK1/2 (Y204) and anti-GAPDH from Santa Cruz Biotechnology (Heidelberg, Germany). The anti-phospho-VEGFR-2 (Y1175), anti-phospho-Src (Y419) and anti-phospho-FAK (Y397) were obtained from Abcam (Cambridge, UK). The rabbit anti-HO-1 was purchased from Enzo Life Sciences (Exeter, UK). All antibodies used in the study are summarised in Table 1. Basement membrane matrix was from BD Biosciences (Oxford, UK). All other cell culture reagents and chemicals, including dimanganese decacarbonyl Mn_2_CO_10_ (CORM-1), tricarbonyldichlororuthenium (II) dimer [Ru(CO)_3_Cl_2_]_2_ (CORM-2), tricarbonylchloro(glycinate)-ruthenium (II) Ru(CO)_3_Cl-glycinate (CORM-3) and sodium boranocarbonate Na_2_(H_3_BCO_2_) (CORM-A1) were obtained from Sigma-Aldrich (Dorset, UK), unless otherwise stated.

**Table 1:** Antibodies used in the study

Antibody name	Molecular weight (kDa)	Phosphorylation site	Final concentration used	Product code
Mouse anti-GAPDH	37	-	0.2 μg/mL	SC-32233
Mouse anti-pERK1/2	42/44	Y204	0.4 μg/mL	SC-7383
Rabbit anti-pVEGFR2	152	Y1175	1.49 μg/mL	Ab-194806
Rabbit anti-pSrc	60	Y419	0.538 μg/mL	Ab-185617
Rabbit anti-HO-1	32	-	1.0 μg/mL	HC3001
Rabbit anti-pFAK	119	Y397	1.0 μg/mL	Ab-81298

### Cell culture

MDA-MB-231, MDA-MB-436 and HECV cell lines were routinely maintained in Dulbecco's Modified Eagle's medium (DMEM/Ham's F-12 with L-Glutamine) supplemented with 10% heat-inactivated foetal bovine serum (FBS) and 1% of antibiotic cocktail mix. MCF-10A cells were routinely maintained in Mammary Epithelial Basal Medium (MEBM, Lonza, Gloucestershire, UK) supplemented with the recommended growth supplements (MEGM kit) (Lonza), 1% antibiotic cocktail mix and 100 ng/mL cholera toxin. HUVEC cells were routinely maintained in Endothelial Cell Growth Basal Medium-2 (EBM-2, Lonza) supplemented with the recommended growth supplements (EGM-2 BulletKit) (Lonza), 1% antibiotic cocktail mix and 2% FBS. All the cells were grown to confluence in 25 cm^3^ or 75 cm^3^ culture flasks loosely capped (Greiner Bio-One Ltd., Gloucestershire, UK) at 37°C in 5% CO_2_ and 95% humidity. The flasks were left to reach adequate confluence before conducting each experiment, unless otherwise stated.

### ELISA for VEGF quantification in supernatant of cell cultures

Upon reaching adequate confluence, the normal media of a cell culture was replaced with fresh media containing the corresponding concentration of CORMs or 1% DMSO or no treatment and the cells were left in the normal incubator for 6 h, 12 h or 24 h. After the indicated incubation time, the supernatant of the flask was collected, centrifuged and aliquoted in 1 mL Eppendorf tubes that were kept at –80°C until required. Protein was also extracted from each flask, quantified using the Bio-Rad DC Protein Assay kit (Bio-Rad laboratories, Hemel Hempstead, UK) and used for normalization of the results. Each aliquot was thawed and used only once and the VEGF content was quantified using the human VEGF ELISA kit (Life Technologies Ltd., Paisley, UK) following the manufacturer's instructions.

### Expression of phosphorylated proteins in VEGF-stimulated HUVEC cells

HUVEC cells were seeded in 6-well plates and upon reaching adequate confluence (~80%), they were serum starved for 12 h. Treatments of 100 μM CORMs or 1% DMSO or serum free media were added to each well and the cells were left in the normal incubator for 15 min. After that, 100 ng/mL VEGF was added for further 5 min. The supernatant of the well was then discarded and the total protein was extracted from each well and quantified. The lysate was used either for ELISA for the quantification of pY1175 following the manufacturer's instructions or for Western blot analysis as reported previously. All the antibodies used in this study are listed in Table 1.

### Conditioned media preparation and tube formation assay

Upon reaching adequate confluence, the normal media of an MDA-MB-231 cell culture was replaced with fresh media containing the corresponding concentration of CORMs or 1% DMSO or no treatment and the cells were left in the normal incubator for 6 h, 12 h or 24 h. After the indicated incubation time, the supernatant of the flask was collected, centrifuged and aliquoted in 1 mL Eppendorf tubes that were kept at –80°C until required. HECV cells were seeded on top of a pre-set layer of Matrigel in each well of a 96-well plate. The plate was left in the normal incubator for cells to adhere and then the full media was replaced with serum free media containing conditioned media from treated MDA-MB-231 cells or direct treatments with CORMs. After 6 h, the wells were imaged using a Leica DM 1000 LED microscope capturing at least 3 images/well in random areas. The images were analysed using the ImageJ software and the percentage of total tube length compared to the wells that received conditioned media from vehicle treated MDA-MB-231 cells or the vehicle treated group was calculated from three independent experiments performed in triplicate.

### Traditional scratch wound assay

HECV cells were seeded in a 24-well plate and left to form a confluent monolayer. The cells were then washed with PBS, wounded with a pipette tip, re-washed twice and treated with normal media containing CORMs or vehicle or no treatment for 24 h. Photos were taken at 0 h, 8 h, 12 h and 24 h after wounding using a Leica DM 1000 LED microscope (Leica Microsystems, Milton Keynes, UK) (5x objective). Migration distances were measured using ImageJ software (National Institutes of Health, NY, USA) and percentage of wound confluence compared to vehicle was calculated from three independent experiments performed in triplicate.

### MTT assay

The MTT assay was conducted as described previously [79]. Briefly, 5 × 10^3^ cells were seeded in normal medium in a 96-well plate and left to attach. The cells were then treated with increasing concentrations of CORMs or vehicle for 72 h. Then MTT solution was added for further 4 h, the medium was removed, and acidified isopropanol was used to dissolve the purple formazan crystals. The absorbance of the test plate was read at 540 nm and an identical blank plate was used to subtract any background absorbance from the test plate absorbance. The absorbance for each compound was subsequently normalized to the vehicle treated cells prior to plotting. Data was statistically analysed using non-linear regression (curve fit) to calculate the IC_50_ value of each compound against each cell line in GraphPad Prism (GraphPad Prism Version 6.0, www.graphpad.com).

### Glycolysis Stress test with the Seahorse Extracellular XF^e^ Flux Analyser

The glycolysis levels of the cells were measured using the Seahorse Extracellular XF^e^ Flux Analyser as reported previously and according to the manufacturer's instructions [80]. Briefly 40,000 cells/well were seeded in a Seahorse XF24 Cell Culture Microplate. The Seahorse Base medium included 1 mM glutamine, was adjusted to pH 7.35 and was kept at 37°C after filtration. The normal media of the cells was removed and Glycolysis media was added before the plate was left in a CO_2_-free incubator for 1 h. All the injections prepared meanwhile were loaded on the injection plate and both plates were inserted and the experiment followed the suggested protocol. After the experiment, a protein quantification assay was performed and the results were normalized to protein concentration/well. The glycolysis levels are presented as x-fold increase in glycolysis rates measured in mpH/min (ECAR) from three independent experiments performed in triplicate.

### HO-1 expression in MDA-MB-231 cells

Upon reaching adequate confluence, the normal media of a cell culture was replaced with fresh media containing the corresponding concentration of CORMs or 1% DMSO or no treatment and the cells were left in the normal incubator for 12 h. After that, the supernatant of the flask was discarded and the total protein was extracted from each flask and subjected to western blotting as reported previously [81]. After the SDS-PAGE, the proteins were transferred onto nitrocellulose membrane which was then blocked and probed with the relevant primary and the corresponding peroxidase-conjugated secondary antibodies. The protein bands were eventually visualized using the chemilluminescence detection system EZ-ECL (Biological Industries, Cromwell, USA). All the antibodies used in this study are listed in Table 1.

### Statistical analysis

Statistical analysis was performed using GraphPad Prism. Unpaired Student's *t-test* with Welch's correction for two groups, non-parametric Mann-Whitney *t-test* for western blot and two-way ANOVA for multiple groups were performed to check for statistical significance, with a *p*-value of <0.05 considered statistically significant. Asterisk notation (^*^) was used to identify significances: ^*^p < 0.05, ^**^p < 0.01 and ^***^p < 0.001.

## SUPPLEMENTARY MATERIALS AND FIGURES


