# Supplementary material for: Repurposing old carbon monoxide-releasing molecules towards the anti-angiogenic therapy of triple-negative breast cancer
Source: Oncotarget. 2019 Feb 1;10(10):1132–48. doi: 10.18632/oncotarget.26638 (PMC6383690; doi:10.18632/oncotarget.26638)
Supplement: Supplementary file 1 [file oncotarget-10-1132-s001.pdf]

Repurposing old carbon monoxide-releasing molecules towards the anti-angiogenic therapy of triple-negative breast cancer

SUPPLEMENTARY MATERIALS

A

IC_{50} (μM)	CORM-1	CORM-2	CORM-3	CORM-A1
**MDA-MB-231**	170.4	116.8	312.5	1529
**MDA-MB-436**	288.8	400.1	358.8	Not converged
**MCF-10A**	387.6	31.87	Not converged	8251
**HECV**	2141	108.3	996.3	327.3

B

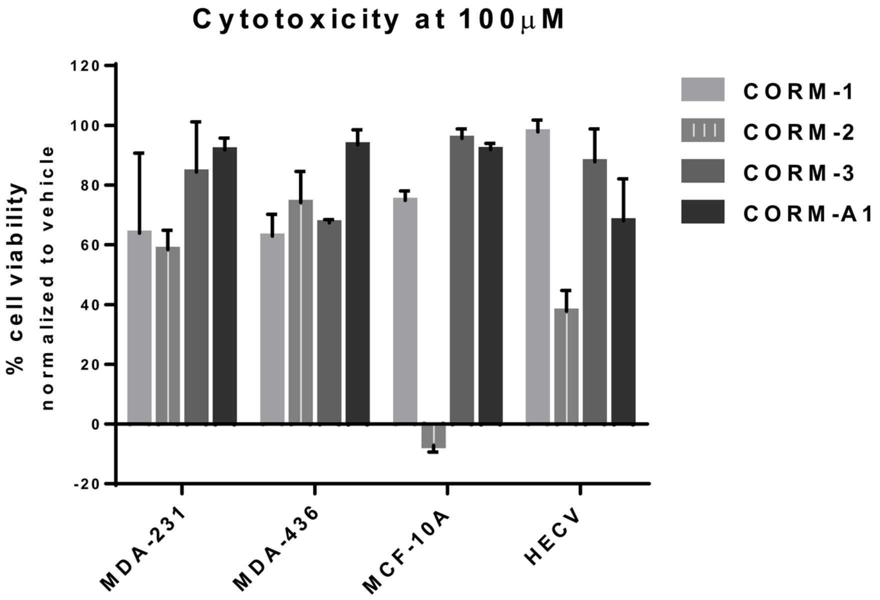

Supplementary Figure 1: Cytotoxicity of CORMs. (**A**) Calculated IC_{50} values for all compounds against the cell lines tested (as calculated in GraphPad Prism). (**B**) Percentage of cell viability for the 100 μM concentration of CORMs (Graph shows average % of cell viability normalized to vehicle +SEM; $n = 3$, $N = 4$).

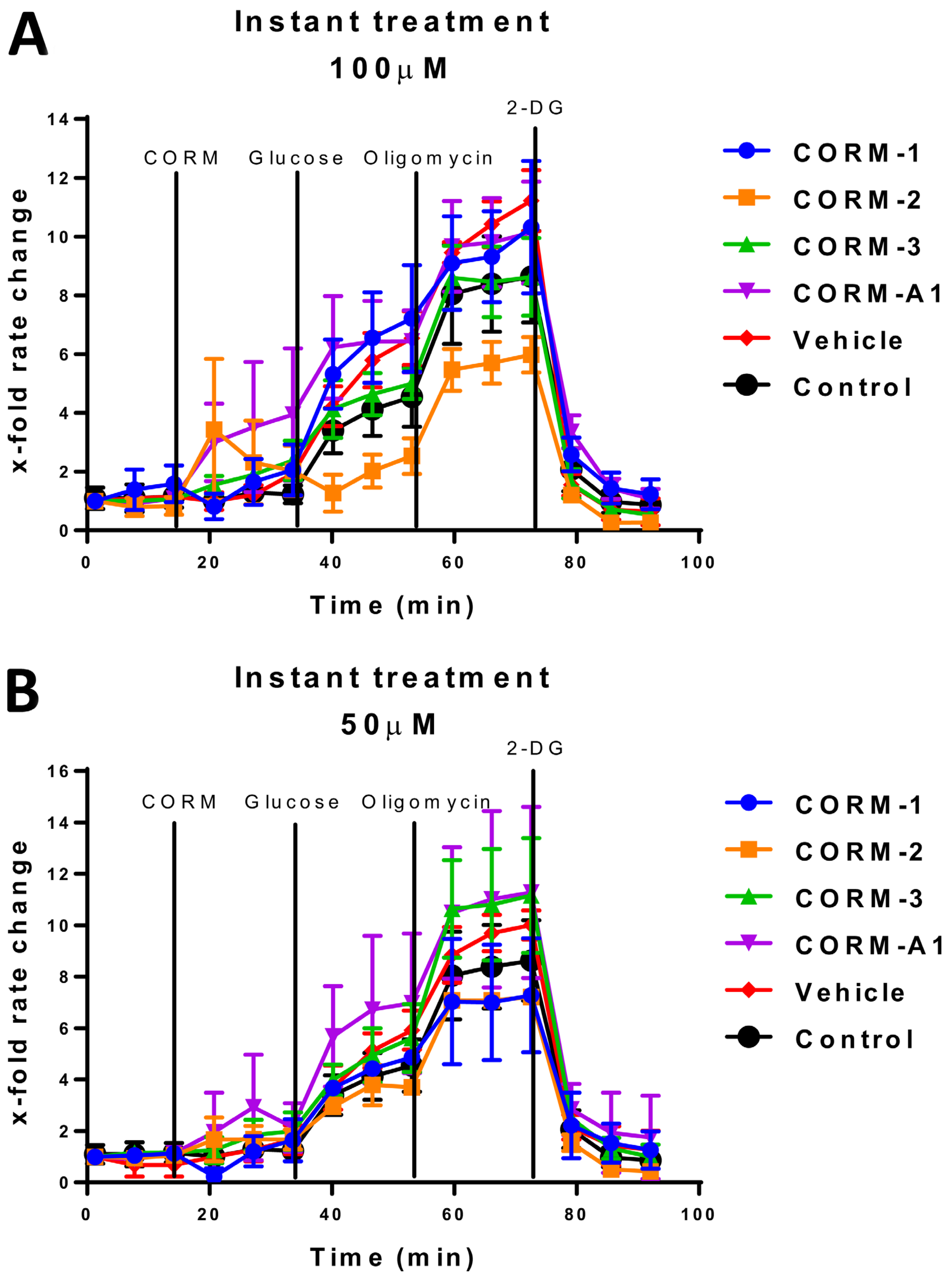

Supplementary Figure 2: Glycolysis stress test in MDA-MB-231 after CORM treatments. Change rate in the glycolysis levels of CORM treated MDA-MB-231 cells, measured as ECAR (mpH/min). (Average ±SEM; $n = 3$, $N = 3$) (All data was statistically analysed against vehicle treated cells using two-way ANOVA: $^{*}p < 0.05$, $^{**}p < 0.01$ and $^{***}p < 0.001$).

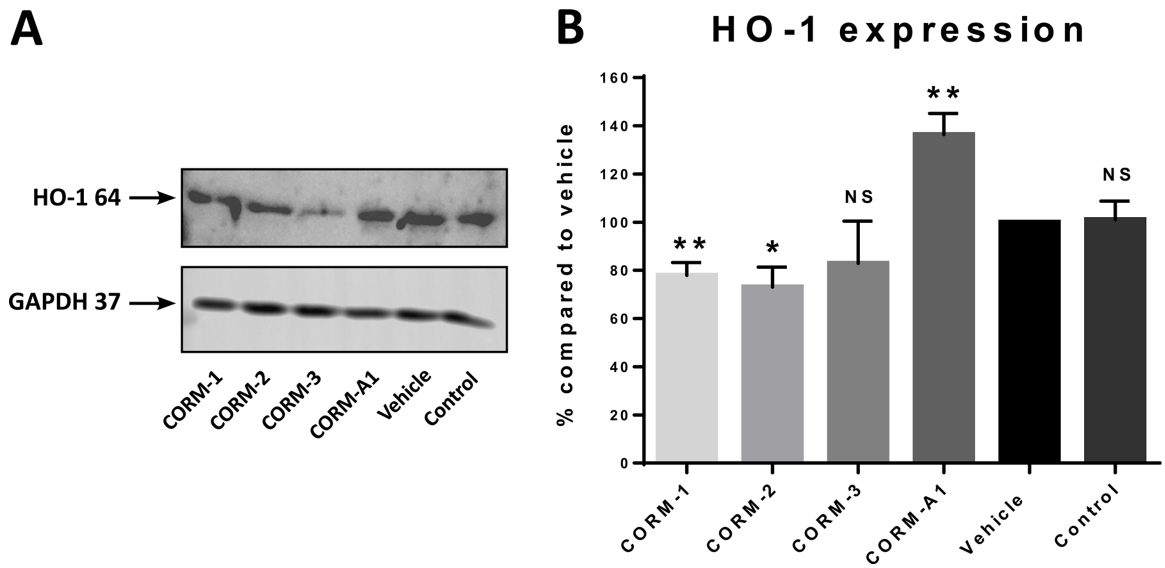

Supplementary Figure 3: HO-1 expression after CORM treatments. (**A**) Western blot of HO-1 expression following CORM or vehicle or media treatments for 12 h in MDA-MB-231 cells. (Blot shows representative data; $N = 4$). (**B**) Assessment of HO-1 levels in MDA-MB-231 cells following 12 h of treatments. (Graph shows % compared to vehicle group +SEM; $N = 4$) (Data statistically analysed using nonparametric (Mann-Whitney) *t*-test with $^{*}p < 0.05$, $^{**}p < 0.01$).
